# Colorectal cancer-derived extracellular vesicles: at the crossroads of the tumor microenvironment and gut microbiota

**DOI:** 10.3389/fimmu.2026.1668737

**Published:** 2026-02-05

**Authors:** Hao Li, Wei Zhang, Wenhui Yan, Kun Wang, Si Chen, Yamao Li, Anzhi Sheng, Anquan Shang, Bingjie Zeng

**Affiliations:** 1 Department of Laboratory Medicine, The Second People’s Hospital of Lianyungang and The Oncology Hospital of Lianyungang, Xuzhou Medical University Lianyungang Second Hospital and Jiangsu University Lianyungang Second Hospital, Lianyungang, China; 2 National Genomics Data Center, Beijing Institute of Genomics, Chinese Academy of Sciences and China National Center for Bioinformation, Beijing, China; 3 School of Future Technology, University of Chinese Academy of Sciences, Beijing, China; 4 Center for Laboratory Medicine, General Hospital of Ningxia Medical University, Yinchuan, China; 5 Department of Central Laboratory, Shanghai Chest Hospital, Shanghai Jiao Tong University School of Medicine, Shanghai, China; 6 Department of Clinical Laboratory, Shanghai Chest Hospital, Shanghai Jiao Tong University School of Medicine, Shanghai, China

**Keywords:** colorectal cancer, extracellular vesicles, tumor microenvironment, gut microbiota, progression, drug resistance, biomarkers, treatment

## Abstract

Colorectal cancer (CRC) remains a leading cause of cancer-related morbidity and mortality worldwide. The clinical treatment faces multiple challenges of significant tumor heterogeneity, prevalence of chemo-resistance, low response rate to immunotherapy, and the impact of the patient’s intestinal microenvironment. Recent studies have shown that extracellular vesicles (EVs), as important information transfer carriers for regulating tumorigenesis and development, play a key role in mediating the complex regulatory network of the gut microbiota-tumor microenvironment (TME). Based on current research advances, our review systematically elucidates how CRC-derived EVs function as dynamic molecular messengers, mediating bidirectional interactions between the TME and the gut microbiota. It also provides a comprehensive outline of EV biogenesis and the key signaling pathways regulated by their diverse molecular cargo. It further delineates how these pathways act in concert to promote the formation of an immunosuppressive microenvironment, drive tumor metastasis, and confer therapy resistance. This review aims to provide a coherent theoretical framework for understanding CRC progression and drug resistance, to offer a scientific rationale for novel therapies targeting CRC-derived EVs, and to highlight future research directions essential for overcoming methodological bottlenecks, deciphering complex interaction networks, and advancing clinical translation.

## 1 Introduction

Colorectal cancer (CRC) ranks among the top causes of cancer-related morbidity and mortality and imposing a substantial burden on healthcare systems worldwide (1, 2). Although surgical resection combined with chemotherapy, targeted and immunotherapy has significantly improved survival in patients, the 5-year survival rate for most patients with intermediate to advanced CRC combined with distal metastases is still below 15% (3). The central therapeutic challenge in CRC lies in tumor heterogeneity and treatment resistance: about 50% of CRC patients develop secondary resistance to oxaliplatin or 5-fluorouracil (4); only 15% of microsatellite instability-high CRC was effective to immunotherapy, and the majority of patients end up with secondary resistance due to the immune-suppressive microenvironment (5, 6). These therapeutic challenges demonstrate that both the progression of CRC and the response to its treatment are not direct outcomes of tumor cell-autonomous genetic alterations, but are instead holistically influenced by persistent and dynamic interactions with the complex surrounding microenvironment (7, 8). Consequently, research on CRC is shifting from a simplistic, tumor cell-centric paradigm toward a more complex framework that views the tumor as an integrated ecosystem (9–11).

Within this ecosystem, dynamic crosstalk among tumor cells, infiltrating immune cells, cancer-associated fibroblasts (CAFs), the extracellular matrix, and vasculature forges a supportive niche pivotal to tumor progression, immune escape, and treatment resistance (12–14). Simultaneously, the vast microbial community residing in the gut—the gut microbiota, often regarded as the host’s “second genome”—exerts profound influence on CRC initiation and progression. It modulates key processes through the secretion of metabolites, regulation of local and systemic immunity, and influence on epigenetic modifications, thereby significantly impacting the efficacy of both chemotherapy and immunotherapy (15–18). A notable observation is the link between enriched Fusobacterium nucleatum (Fn) and worse CRC prognosis. The population-level variance in this association highlights that the functional impact of the gut microbiota is not uniform, thereby supporting a highly context-dependent model of host-microbe interaction (19). Although the tumor microenvironment (TME) and the gut microbiota are anatomically and conceptually distinct, their functions are profoundly intertwined. Consequently, how these two systems achieve dynamic bidirectional crosstalk to cooperatively shape a systemic state conducive to tumor progression and therapy resistance remains a pivotal, unresolved question in the field.

Recent studies have identified extracellular vesicles (EVs) as critical information carriers that facilitate communication across cells and organs, potentially serving as key molecular messengers in mediating this cross-domain interaction (20). Serving as key mediators of intercellular communication, EVs are nanoscale, lipid-bilayer particles released by cells. They function by carrying proteins, nucleic acids, and lipids, thereby weaving a complex communicative web between tumor cells, stromal cells, immune cells, and the gut microbiota (16, 21–23). Evidence indicates that tumor-derived EVs may play an active role in remodeling the TME. Specifically, it has been found that LC3-dependent EVs secreted by CRC cells induced an immunosuppressive phenotype in myeloid-derived suppressor cells (MDSCs) through the TLR2-MYD88 pathway, leading to aberrant secretion of IL-10 and arginase-1 (24). However, these findings must be interpreted with caution: the conclusions are drawn primarily from *in vitro* co-culture models, and the EV preparations used likely constitute heterogeneous populations. Their physiological relevance therefore requires validation within more complex *in vivo* settings. On the other hand, emerging evidence indicates that the composition and state of the gut microbiota can significantly modulate the secretion of EVs from CRC cells and influence the loading of their molecular cargo, thereby amplifying and relaying these microbial signals to the TME (25). As one of the drivers of regulation, EVs in the complex network of the TME may be key vectors mediating the dialogue between the gut microbiota and the TME (26, 27). The signaling between the TME and gut microbiota, mediated by EVs, constitutes a dynamic, bidirectional network. The reciprocal communication mechanism itself presents a potential strategic target for dismantling immunosuppressive barriers (28). However, to achieve precise intervention in this network, it is essential to first decipher its complex remodeling under therapeutic pressure. Beyond the PD−1/PD−L1 axis, the significance of the CTLA−4 pathway is growing. Tumor−derived EVs contribute to this axis by shaping antigen−presenting cell function and amplifying regulatory T cells, thereby enhancing CTLA−4−mediated suppression, which provides a novel perspective on the limited efficacy of CTLA−4 monotherapy (29–31). Furthermore, chemotherapy (e.g., capecitabine) exhibits a well−recognized dual immunomodulatory nature. While it can induce immunogenic cell death and “prime” anti−tumor immunity, the therapeutic pressure it exerts simultaneously triggers the release of “stress−induced EVs” from tumor cells. These EVs are enriched with immunosuppressive molecules such as PD−L1 and non−coding RNAs. Acting as pivotal mediators, they disseminate adaptive resistance signals within the TME and, in synergy with chemotherapy stress, collectively drive the activation of key inhibitory pathways including PD-1/PD−L1 and CTLA−4 (32). More importantly, a profound dynamic interplay exists between therapy−induced stress−driven EV remodeling and the ongoing immunomodulatory influence exerted by the gut microbiota via EVs, which together constitute a dynamic, multi−layered adaptive immune−resistant ecosystem (33–35). Therefore, revealing the dynamic regulatory network of EVs in TME and investigating their specific mechanisms driving the evolution of tumor heterogeneity and treatment resistance will provide a new perspective on clinical diagnostic and therapeutic strategies for CRC.

Nevertheless, the EV research field faces formidable methodological and conceptual challenges. High vesicle heterogeneity, imperfect isolation methods yielding mixed preparations, and the prevalent use of simplistic *in vitro* systems together obscure definitive functional attribution and cast doubt on the physiological relevance of observed mechanisms. Therefore, a critical appraisal of both the strength of available evidence and the limitations of methodological approaches is essential when interpreting the functions of EVs in CRC, particularly in mediating gut microbiota-TME interactions. Accordingly, this review aims to elucidate these challenges in EV research, with a focus on the EV-mediated “gut microbiota-TME” network, to examine its translational potential and practical hurdles as novel diagnostic biomarkers and therapeutic targets, and to provide a theoretical framework with a comprehensive and critical perspective on CRC.

## 2 Biological properties and functions of EVs

The development and treatment resistance of CRC involves dynamic remodeling of the TME and aberrant regulation of intercellular communication (36). In recent years, EVs have become a hot direction of research by delivering functional molecules to remodel TME, mediating multiple mechanisms such as tumor progression and therapeutic resistance, and creating a vicious circle. However, any interpretation of the roles played by EVs requires careful consideration of the inherent complexities and methodological challenges within this field of research.

### 2.1 Biogenesis, heterogeneity, and methodological foundations of EVs

**Figure 1:**
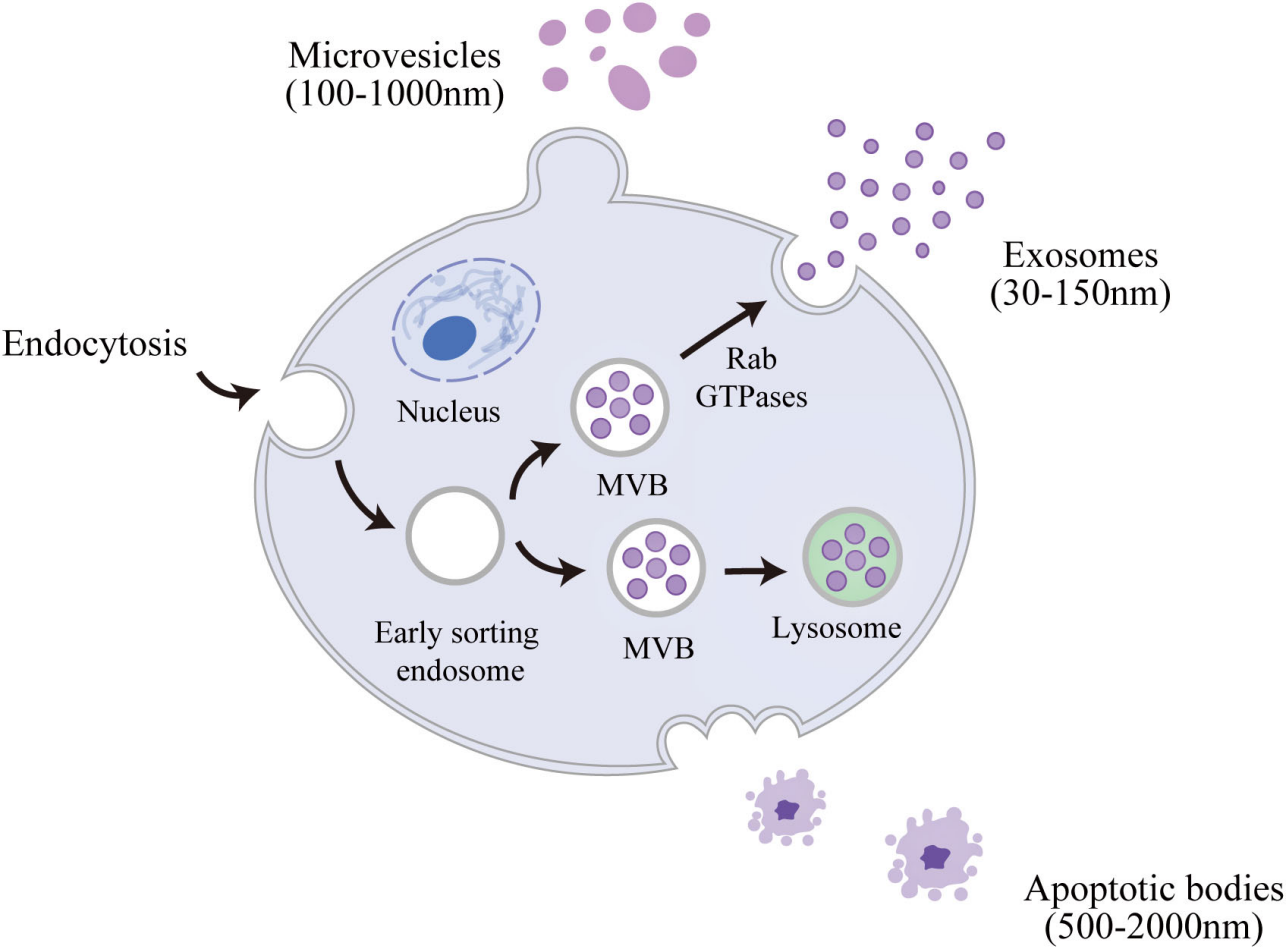
Biogenesis and secretion release of Cellular EVs: exosomes (30–150 nm), microvesicles (100–1000 nm) and apoptotic bodies (500–2000 nm).

EVs are a class of nanoscale membrane vesicle structures actively secreted by cells, which can be classified into three major categories based on their biogenesis and size: exosomes (30–150 nm), microvesicles (100–1000 nm) and apoptotic bodies (500–2000 nm) (37, 38). The generation of EVs is a highly regulated process: exosomes are formed and released via the endosomal sorting complex required for transport-dependent/non-dependent pathway in the endosome-multivesicular bodies pathway; microvesicles are generated by direct outgrowth and shedding via the plasma membrane; and apoptotic vesicles are a specialized subclass of cells that are formed by the plasma membrane wrapping intracellular debris in late stages of apoptosis (39) (**Figure 1****).** These nanoscale vesicles are widely found in biological fluids such as blood, cerebrospinal fluid, urine and saliva, and constitute a complex intercellular bioinformatic network by delivering functional proteins, lipid molecules and nucleic acids, thereby playing crucial roles in both maintaining physiological homeostasis and driving disease pathogenesis (40, 41).

Nevertheless, the limitations inherent in current research methods represent an inescapable central issue. The continuously updated MISEV guidelines from the International Society for EVs authoritatively state that EVs constitute a heterogeneous family encompassing multiple subtypes, including exosomes, microvesicles, and apoptotic bodies (42–44). Currently, the vast majority of published functional studies on tumor-derived EVs rely on preparations obtained through methods such as differential ultracentrifugation, size-exclusion chromatography, polymer-based precipitation, or antibody-based capture: differential ultracentrifugation and density gradient centrifugation separate EVs based on their buoyant density (45, 46); size-exclusion chromatography and ultrafiltration exploit differences in particle size compared to other contaminants (47–49); while antibody capture methods isolate EVs according to their biochemical characteristics (50). Although these methods are standard, none yields absolutely pure EV subsets. This fundamental limitation makes it challenging to definitively assign a given biological function, such as miRNA-mediated activity, to a specific EV subtype. Consequently, it is imperative to cautiously evaluate the purported functions of EVs with careful consideration of the methodology used for their isolation and characterization. To address this challenge, the latest MISEV guidelines strongly recommend multimodal characterization of EV preparations. This includes morphological assessment by transmission electron microscopy, determination of particle size distribution and concentration via nanoparticle tracking analysis, and detection of EV-positive (CD9, CD63, CD81) and EV-negative (Calnexin, ApoB) markers by western blotting (44). Such comprehensive profiling is essential for accurately defining the study material and enhancing experimental reproducibility. Therefore, any discussion of purported EV functions must acknowledge that conclusions are derived from heterogeneous preparations obtained through specific methodologies, and that mechanistic interpretations are inherently framed by the particular scientific context.

Moreover, EV heterogeneity extends beyond physical size to encompass their cellular origins. EVs derived from different sources exhibit marked functional diversity, with the biological functions of EVs from different sources showed significant heterogeneity, and the differences in their roles were mainly attributed to a variety of factors, such as the molecular components they carried, the tissue microenvironment in which they were located, and the characteristics of the target cells (51).Current studies have revealed that the mechanism of action of EVs mainly includes specific binding of ligands on the surface of EVs to target cell membrane receptors, direct delivery of bioactive molecules by membrane fusion between EVs and target cells, and transmembrane signaling mediated by endogenous signaling molecules released by EVs interacting with target cell surface receptors (38, 52). Some studies have previously reported that cells cultured *in vitro* can also exhibit active EVs secretion characteristics (53, 54). The secreted EVs are able to trace the biological characteristics of the cells from which they originate, mediate intercellular signaling, and play a regulatory role in the maintenance of normal physiological functions of the organism and in the development of diseases (41). Particularly in tumor patients, these nanoscale vesicles trigger effective responses such as inhibition of anti-tumor immunity (55, 56), remodeling of the TME (57, 58) and induction of tumor angiogenesis (59, 60) through multidimensional regulatory mechanisms. Dysregulated expression of Rab GTPase family proteins and SNARE complexes within tumor cells has been observed to promote EV secretion, which correlates with increased tumor aggressiveness in CRC (61, 62). However, this correlation remains contingent upon the appropriate isolation and definitive characterization of the EV preparations used.

### 2.2 Molecular function of CRC-derived EVs

EVs, as important signaling mediators in TME, play a central role in the communication among tumor cells, immune cells, and stromal cells. Studies have shown that sEVs secreted by CRC cells can affect the phenotype and function of recipient cells through multiple mechanisms, such as down-regulating the PTEN/AKT/NF-κB pathway through the delivery of miRNA-372-5p and inducing PD-L1 expression to inhibit T-cell activity, thereby creating an immunosuppressive microenvironment (63). Nevertheless, a cautious interpretation of this mechanism is warranted: the study is primarily based on *in vitro* co-culture models, and the EVs used for functional validation are heterogeneous preparations isolated via conventional ultracentrifugation. Their physiological relevance, particularly the extent to which CRC-derived EVs rely solely on this specific pathway to dominate T−cell suppression within the complex *in vivo* immune milieu, requires more rigorous validation. Interestingly, Rezaei’s team demonstrated that CRC-derived exosomes enhance anti-tumor immune responses by delivering miR-124-3p (64). Such discrepancies in conclusions are likely attributable to differences in experimental models, variations in the molecular cargo of EV preparations, or distinct immune-modulatory patterns inherent to specific molecular subtypes of CRC. This clearly indicates that the immunoregulatory functions of EVs are not unidirectional but exhibit marked context-dependence and complexity.

Additionally, it should be noted that EV-mediated immunosuppression is a multi-target, networked process. CTLA-4 is another critical immune checkpoint that exerts inhibitory effects during the early stages of T cell activation and also plays a significant role within the immunosuppressive microenvironment of CRC (65). EVs may modulate the CTLA−4 pathway directly or indirectly via the delivery of signaling molecules, the modulation of antigen−presenting cells (APCs), the induction of regulatory T cells (Tregs), and the remodeling of myeloid cells (29, 66, 67). Particularly in microsatellite-stable (MSS) type, CTLA-4 pathway-mediated immunosuppression is a key mechanism underlying the TME phenotype of “immune cell infiltration coupled with functional impairment” in CRC (68). Furthermore, through high expression of CTLA−4, Tregs mediate “trans−endocytosis” to strip CD80/CD86 from the surface of APCs, thereby physically blocking the co−stimulatory signals required for naïve T−cell activation (69). Concurrently, CTLA−4 signaling curbs excessive mTOR activation within Tregs themselves, maintaining their survival and phenotypic stability to ensure persistent immunosuppressive function (32, 70). The limited efficacy of CTLA−4 monotherapy in unselected CRC patient populations may stem, in part, from tumor−derived EVs, which deliver immunosuppressive signals to establish and dynamically sustain an inhibitory network centered on the Treg/CTLA−4 axis, thereby creating critical, multi−layered barriers to therapy.

Within the dynamic remodeling of the TME, EVs exhibit pronounced spatial heterogeneity and functional diversity. CRC-derived small EVs enriched with miR-21-5p and miR-200a, which can induce M2 polarization in macrophages and upregulate their PD-L1 expression, thereby inhibiting the function of CD8^+^ T cells (71). In addition to nucleic acid cargo, proteins displayed on the EV membrane surface possess direct functional roles. Studies have shown that tumor-derived exosomes can excessively activate cholesterol and phospholipid metabolism in T cells via their carried PD-L1, leading to lipid accumulation which subsequently induces T cell senescence and functional exhaustion (72). Furthermore, studies under more physiologically relevant *in vivo* conditions have also investigated EV functions. For example, analysis of a CRC liver metastasis model revealed that tumor cell-secreted exosomes enriched with miR-1246 can be taken up by hepatic stellate cells (HSCs), and these exosomes target the INSIG1 gene and activate SREBP2-mediated cholesterol metabolic reprogramming, which in turn promotes HSC activation via the TLR4/NF-κB/TGF-β pathway, ultimately driving the formation of a pro-metastatic microenvironment (73). This work provides higher-level evidence that functionally links molecular mechanisms identified *in vitro* with pathological phenotypes observed *in vivo*.

Meanwhile, the sustained proliferation and metastasis of CRC are closely associated with immune escape mechanisms, where tumor-derived EVs escape innate immune surveillance by delivering bioactive molecules and shaping immunosuppressive ecological niches in the TME (26). EV-mediated immunoregulation constitutes a multi-pathway, networked process, in which long non-coding RNAs (lncRNAs) serve as one class of important mediators. Exosomes from CRC cells mediate immunosuppression via transfer of lncRNA KCNQ1OT1, which elevates USP22 levels by sponging miR-30a-5p, ultimately resulting in PD-L1 protein stabilization and diminished CD8^+^ T cell responses (74). And exosomes carrying lncRNA SNHG10 promote INHBC expression and activate the TGF−β pathway, thereby suppressing the cytotoxicity of natural killer cells (75). In addition, EGFR-mutant CRC cells can utilize EVs to transfer a distinct set of lncRNAs into the TME, which systematically promotes the establishment of an immunosuppressive microenvironment (76). In synthesizing these findings, we must confront a persistent debate in the field: the copy numbers of functional nucleic acids, such as miRNAs or lncRNAs, within individual EVs are typically very low. Whether this low abundance is sufficient to mediate significant gene-regulatory effects on recipient cells at physiological concentrations remains a subject of debate. Some scholars posit that EVs may overcome this limitation through high-frequency and sustained signaling or by the coordinated action of multiple cargo molecules. Alternatively, certain highly enriched proteins or lipids within EVs might serve as the dominant functional effectors. While EV-mediated immunomodulation offers novel insights into CRC therapy resistance and potential interventions, and while these collective findings provide a theoretical basis for precision diagnostics based on exosomal signatures, future research must focus on precisely quantifying EV cargo and validating its biological activity at physiologically relevant doses. Although the immunoregulatory mechanism of EVs provides a new perspective for understanding the therapeutic resistance and intervention of CRC, and all of these discoveries lay a theoretical foundation for the development of precision diagnostic strategies based on the molecular characteristics of EVs. Future research requires more precise quantification of EV cargo and validation of their functions at physiologically relevant doses.

### 2.3 Early roles of EVs in colorectal carcinogenesis

Having elucidated the molecular functions of CRC-derived EVs in intercellular signaling, we now focus on their potential roles in the initiation and early promotion of CRC, examining this through the lens of established carcinogenesis models. Examining EV biology through these lenses provides an analytical framework to evaluate their temporal and functional roles in tumor initiation and early promotion.

The conventional adenoma-carcinoma sequence, accounting for approximately 60-70% of CRC cases, is a genetically ordered, multi-step process initiated by the inactivation of the APC tumor suppressor gene (77–79). This seminal event leads to constitutive activation of the Wnt/β-catenin pathway, deregulated crypt homeostasis, and the formation of precancerous adenomas (80, 81). Subsequent acquisitions of mutations in KRAS and TP53, among others, drive the progression towards invasive carcinoma (82). EVs derived from pre-neoplastic lesions may contribute to the establishment of a “field cancerization” microenvironment from the earliest stages. By mediating paracrine signaling, they participate in the molecular preconditioning of histologically normal tissue, thereby facilitating the subsequent clonal expansion of transformed cells (83).

Although direct evidence from early human adenomas remains limited, data from experimental models and observational human studies provide growing support for this idea. For instance, EVs isolated from the plasma of patients with colorectal adenomas show distinct molecular profiles compared to healthy controls, including enrichment of specific lncRNAs and mRNAs that distinguish adenoma cases from healthy individuals (84). Furthermore, studies have reported that tumor-derived sEVs can activate the ERK signaling pathway in normal epithelial cells, leading to the upregulation of the oncogene CREPT, while concurrently initiating an inflammatory response via the TNF pathway. The upregulated CREPT further modulates the expression of TNFR2 and PI3K, thereby mediating a regulatory loop that amplifies and sustains oncogenic inflammatory signaling to ultimately drive malignant transformation (85). Concurrently, CRC-derived EVs remodel the TME by delivering specific molecular cargo such as the CDC42 protein to macrophages, which activates the intracellular NOD1 signaling pathway in these immune cells to promote pro-inflammatory responses and enhance phagocytic function (86). CRC-derived EVs also activate CAFs, stimulating the secretion of pro-inflammatory and pro-tumorigenic factors and thus fostering a microenvironment conducive to early carcinogenesis (87). Collectively, The molecular events and microenvironmental alterations mediated by EVs represent not only pivotal early drivers in CRC pathogenesis but also a promising source of biomarkers, as the specific molecules they carry hold significant potential for early diagnosis and risk assessment of CRC.

However, it must be emphasized that direct experimental evidence causally linking specific EV cargo to the functional evolution of defined pre-neoplastic lesions is currently limited. Most studies have focused on EVs from established cancer cell lines or advanced tumors. To definitively answer whether EVs are initiators or accelerants, future research must employ more physiologically relevant models of early carcinogenesis. This includes longitudinal studies using organoids derived from patient-matched normal mucosa, adenomas, and carcinomas to profile EV secretion and cargo dynamics across stages. Furthermore, genetically engineered mouse models of intestinal tumorigenesis combined with EV depletion or tracking strategies will be crucial to establish the *in vivo* necessity and sufficiency of EVs in the adenoma-carcinoma sequence. Addressing these gaps will not only clarify the temporal role of EVs in CRC pathogenesis but also open novel avenues for intercepting cancer development at its earliest, potentially reversible stages through EV-targeted strategies.

### 2.4 Relationship between EVs and malignant progression of CRC

**Figure 2:**
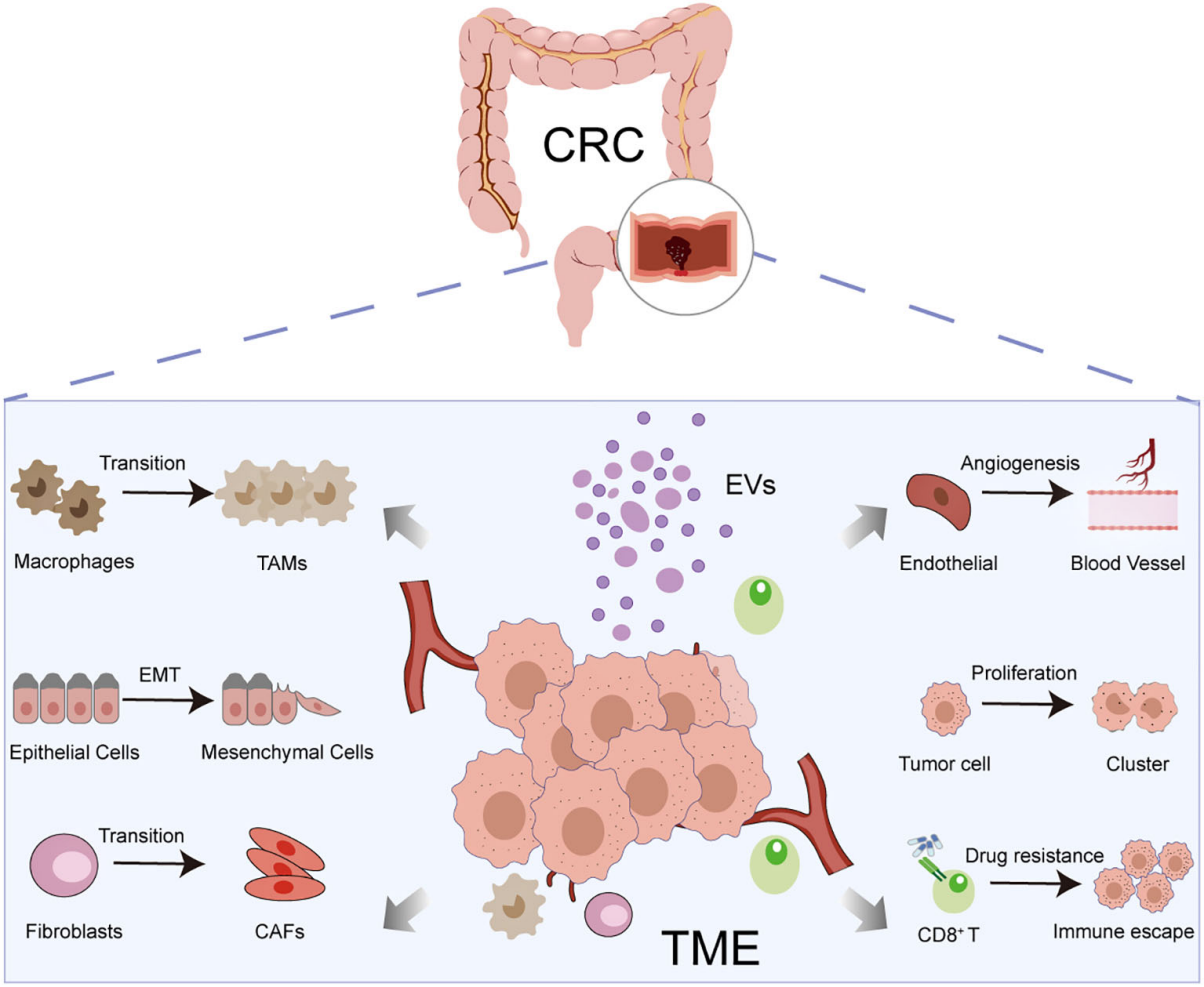
The core functional roles of EVs in the TME: EVs modulates the TME via immune suppression, angiogenesis, cellular transformation, and drug resistance in CRC.

Following the establishment of a tumor, EVs emerge as pivotal mediators within the TME, playing a well-characterized role in driving malignant progression and therapeutic resistance (86, 88). EVs achieve transcellular regulation by delivering functional molecules that promote tumor progression and immune evasion, thereby directly fueling CRC invasion, metastasis, and therapy resistance, and permeating multiple facets of malignant progression (**Figure 2**).

During the development of CRC, tumor cells can form a microvascular network through neovascular sprouting to support their rapid proliferation and spread, and EVs are key regulatory mediators of the tumor angiogenesis process (89–91). It has been shown that exosomal miR-1229 derived from serum of CRC patients mediates the upregulation of vascular endothelial growth factor (VEGF) expression through inhibition of HIPK2, which in turn drives human umbilical vein endothelial cell neovascularization in ex vivo and *in vivo* models (92). Research in xenograft mouse models further demonstrates that CRC−derived circular RNA circCOL1A1 is transported via exosomes and, upon binding to EIF4A3 in target cells, triggers the Smad2/3 phosphorylation cascade, thereby inducing the secretion of pro−angiogenic factors and promoting pathological tumor angiogenesis (93). Notably, some studies have reported that CRC-derived EVs can suppress anti-tumor immune responses by carrying specific molecules that mediate the dialogue between tumor cells and tumor-associated macrophages (TAMs) (94). During the metastatic cascade, EVs serve as critical messengers in establishing the pre−metastatic niche. CRC cells undergoing epithelial−mesenchymal transition (EMT) deliver miR−106b−5p via exosomes, thereby inducing M2 polarization in macrophages (95). It was found that TAMs in the TME of patients with CRC liver metastases could promote the formation of the pre-metastatic niche (PMN) of CRC by down-regulating PTEN expression via exosomal delivery of miR-934 and by activating the PI3K/AKT signaling pathway to induce the polarization of M2-type macrophages (96). These “educated” macrophages, in turn, enhance the EMT and migratory capacity of CRC cells through secreted factors, thereby establishing a self−reinforcing malignant positive−feedback loop. Similarly, highly metastatic CRC cells secrete exosomes enriched with miR−106a−5p, which induce M2−type macrophage polarization, thereby suppressing T−cell function and promoting liver metastasis (97). Transfer-initiated CTC-derived EVs play a key role in cancer progression and spread by mediating sustained bidirectional crosstalk between tumor cells and the TME (98).

In addition, EVs not only play a pivotal role in tumor progression and metastasis, but are also key drivers in mediating resistance to chemotherapy, targeted therapy and immunotherapy (99) (**Table 1**). EVs serve as pivotal mediators of tumor therapy resistance. They directly expel chemotherapeutic drugs via exocytosis or transfer detoxifying molecules, such as glutathione S-transferases, thereby reducing the effective intracellular drug concentration or activity (100). Cancer cells actively deliver drug resistance-associated molecules to target cells through secretion of EVs, which remodels the physicochemical properties and signaling network of TME to form an adaptive drug resistance microenvironment (28). As carriers of intercellular communication, these EVs play a crucial role in shaping the tumor immune microenvironment to mediate adaptive resistance. Chemotherapy, particularly with fluoropyrimidines such as capecitabine, is a cornerstone of CRC treatment. It orchestrates a “priming” signal for anti-tumor immunity by inducing immunogenic cell death, enhancing tumor antigen exposure, and transiently depleting immunosuppressive populations like MDSCs (101). However, this potential immunostimulatory effect is often counteracted by a treatment-induced, tumor-driven adaptive response. Under stress, tumor cells markedly increase the release of EVs laden with immunosuppressive cargo. These vesicles may carry immune checkpoint ligands, regulatory RNAs, and metabolism-altering enzymes (102–104). Acting as pivotal intercellular messengers, EVs broadly disseminate tumor-derived stress signals throughout the TME, which thereby suppresses T cell function and ultimately fosters adaptive resistance to both the initial chemotherapy and subsequent immunotherapies. Consequently, a highly dynamic and paradoxical interaction exists between chemotherapy and immune checkpoint pathways. Chemotherapy-induced cellular stress can upregulate the expression of immune checkpoints, including the PD-1/PD-L1 and CTLA-4 axes, creating a microenvironment of “activation followed by suppression”. EVs serve as a key medium for transmitting this stress-adaptation signal, contributing to the limited benefit of sequential therapies (105–107).

**Table 1:** CRC-derived EVs regulation of the tumor.

Target cell	Cellular/process transition	EV-mediated role and mechanism	Key molecules/signaling pathways	References
Macrophages	Transition to TAMs	CRC-derived EVs carry specific molecules that induce macrophage polarisation towards the TAMs, thereby suppressing anti-tumour immunity and promoting metastasis.	miR-934/PTEN/PI3K/AKT miR-106a-5p miR-106b-5p/PDCD4/PI3Kγ/Akt/mTOR	(95–97)
Tumor Cell	Proliferation	CRC-derived EVs playing a crucial role in the survival, proliferation, and distant seeding of tumor cell clusters.	–	(98)
Endothelial Cells	Angiogenesis	CRC-derived EVs transfer pro-angiogenic signals to TME, driving their aberrant proliferation and angiogenesis to nourish tumor growth.	miR-1229 / HIPK2 / VEGF circCOL1A1 / EIF4A3 / Smad2/3	(92, 93)
CD8^+^ T Cells	Drug resistance	CRC-derived EVs deliver miRNAs that directly target and inhibit T-cell co-stimulatory molecules, preventing effective T-cell activation.	miR-424 / CD28, CD80	(109)

Furthermore, drug−resistant cells can transmit pro−survival signals to sensitive cells via EVs. For instance, under the selective pressure of BRAF inhibitors, CRC cells harboring the BRAF V600E mutation package feedback signals such as EGFR into EVs. Upon delivery, these signals enable recipient cells to circumvent drug inhibition, ultimately leading to targeted therapy failure (108). Fortunately, researchers have developed a nucleic acid drug design based on EVs-siBRAFV600E, which can specifically inhibit the growth and metastasis of this mutant CRC, showing promise as a novel therapeutic strategy for patients. CRC-derived EVs can bind to the αvβ3 integrin on macrophages via surface MFGE8 protein, activating the intracellular Src-FAK-STAT3 pathway, thereby enhancing the phagocytic clearance of apoptotic cells by macrophages and ultimately impairing the efficacy of cisplatin-based chemotherapy (99). Furthermore, it has been found that EVs secreted by CRC cells can inhibit T-cell activation by targeting T-cell co-stimulatory molecules CD28 and CD80 via miR-424, assisting the tumor to undergo immune escape thus leading to ineffective treatment with immune checkpoint inhibitors (ICIs) (109).

The evidence summarized establishes several mechanistic routes for EV-driven tumor progression and therapy resistance. However, tumor aggressiveness and treatment failure are systemic outcomes arising from intertwined genetic, epigenetic, metabolic, and microenvironmental perturbations. Within this network, EVs function more as central communication nodes and phenotypic amplifiers, integrating multifaceted signals derived from chemotherapy-induced stress and immune checkpoint regulation (110). In particular, a deeper understanding of PD−1/PD−L1 and CTLA−4−mediated immunoregulation, an analysis of the immunological consequences of capecitabine−based chemotherapy, and insights into EV−driven resistance mechanisms together constitute a more coherent framework for optimizing immunotherapy strategies in CRC (65, 111, 112). Therefore, it is essential to adopt an integrated systems biology framework to fully decipher their role, moving beyond an isolated view. Equally important is the consideration of the “dose effect” when evaluating experimental evidence. Many convincing mechanisms were identified using high or enriched EV doses. It remains unclear whether the actual concentrations of EVs in circulation or local niches under physiological or pathological conditions are sufficient to initiate the same cascades. This gap requires validation through kinetic studies employing physiological doses. Furthermore, current research models are inherently limited. Many conclusions rely on *in vitro* co-culture systems involving a single cell type or on xenograft models in immunodeficient hosts. These models fail to fully recapitulate the integrated immune microenvironment, tissue architecture, and cellular diversity of human CRC *in vivo*. Future translational research should aim to develop models that better recapitulate the complexity of human disease and to explore the integration of EV-based biomarkers—such as specific immunosuppressive cargo—into clinical trials. These biomarkers should guide optimal timing and patient selection for therapies combining chemotherapy, ICBs (targeting PD-1/PD-L1 and CTLA-4), and EV-targeting strategies. Consequently, advancing EV-targeted strategies toward clinical translation requires a clear acknowledgment of this biological complexity and the accompanying technical challenges, coupled with a committed effort to develop models that more faithfully mirror human disease pathophysiology.

## 3 Association of gut microbiota with CRC

The development of CRC is a multifactorial process involving a complex interaction of genetic, epigenetic modifications and environmental factors. The gut microbiota, a dense and resident microbial ecosystem, constitutes a pivotal environmental component that interfaces the host with its milieu. It integrates into the complex regulatory circuitry of CRC via multifunctional mechanisms encompassing metabolism, immunity, and epigenetic regulation (9, 113, 114). In recent years, a large number of studies have confirmed that intestinal flora dysbiosis is closely related to the development of CRC.

### 3.1 Gut microbiota affects the development of tumor

The ongoing and dynamic interplay between the gut microbiota and the host can exert a profound influence on the initiation and progression of CRC through direct and indirect epigenetic regulation, with large-scale population studies providing macro-level evidence for this relationship. Metagenomic analyses of a multicenter cohort showed a significant reduction in the alpha diversity of the gut microbiota in CRC patients, with an elevated abundance of oncogenic bacteria and a reduction in short-chain fatty acid-producing commensal bacteria (115, 116). To explore the potential causality of this association, a Mendelian randomization study revealed that thirteen gut microbiota were significantly associated with CRC risk, four of which were found to mediate the causal relationship between immune cells and CRC (117). Collectively, these population-based data provide robust support for the instrumental role of specific microbial taxa in the development and progression of CRC.

At the mechanistic level, specific bacteria and their metabolites can act via multiple pathways to directly or indirectly drive tumorigenesis. Researchers first identified a unique pattern of DNA damage caused by human gut microbiota on CRC in 2020 (118). Specifically, dysbiosis of the gut microbiota can induce colorectal carcinogenesis by directly damaging intestinal epithelial DNA through metabolites such as butyrate and secondary bile acids or through the activation of pattern−recognition receptors like TLR4, which initiates NF−κB−dependent chronic inflammation, ultimately promoting a cancer−favorable microenvironment (119). Recent studies have shown that disruption of gut microbiota by antibiotics leads to upregulation of the lncRNA Snhg9 and accelerates the progression of CRC (120). And the bifidobacterium fragilis toxins (BFT) secreted by enterotoxigenic bacteroides fragilis (ETBF) disrupts the intestinal epithelial barrier and induces IL-8 release, leading to sustained activation of the STAT3/NF−κB signaling pathway. This not only triggers inflammation but also causes aberrant DNA methylation, thereby allowing ETBF to function as a “tumor initiator” (121–124).Parvimonas micra (P. micra) induces oxidative stress and mitochondrial dysfunction by secreting hydrogen sulfide and promotes malignant cell transformation (125). Furthermore, the gut microbiota can modulate host metabolic pathways such as the urea cycle and establish an “immune-microbial metabolic axis”, thereby systemically reprogramming the host’s immunometabolic state and influencing susceptibility to CRC (113). It is important to emphasize that although these mechanisms are strongly supported by animal models and *in vitro* experiments, validating their causal chronology and contribution magnitude in humans remains challenging. Moreover, human gut microbiota composition is influenced by diverse factors such as diet, geography, and antibiotic use, leading to variations in the abundance of specific bacteria and the strength of their associations across different population studies. Therefore, these limitations must be considered when interpreting epidemiological data and designing intervention strategies.

### 3.2 Influence of gut microbiota on TME

A distinctive feature of advanced CRC is a profoundly immunosuppressive microenvironment, which is not only a consequence of tumor progression but also a key driver of treatment resistance (126). The gut microbiota shapes the immune phenotype of TME through a continuous dialogue with the host immune system via pattern recognition receptors (127). Moreover, gut microbiota profoundly influences CRC development and therapeutic response by modulating immune cell differentiation, cytokine secretion and metabolic pathway activation in the TME (128, 129). With the advancement of multi-omics technology, the mechanism of the role of gut microbiota and its metabolites in the immune regulation of CRC has been gradually revealed. For example, the microbial metabolite 4-hydroxyphenylacetic acid activates the JAK2/STAT3 pathway in myeloid cells, induces CXCL3 expression, recruits immunosuppressive PMN-MDSCs into the TME, and thereby suppresses the antitumor function of CD8^+^ T cells (130). In addition, phenylacetylglutamine, a metabolite of gut microbiota origin, was found to be strongly associated with the efficacy of ICIs. Mechanistically, it can directly suppress CD8^+^ T cell function by activating the adrenergic receptor signaling pathway, thereby contributing to resistance to anti−PD−1 therapy. Clinically, higher levels of phenylacetylglutamine (PAGln) are associated with reduced progression−free survival in patients (131). These findings reveal that microbiota-derived metabolites function as systemic signaling molecules to regulate immune responses within the TME.

Moreover, there was a spatial heterogeneity in the regulation of TME by the gut microbiota. On one hand, bacteria can colonize the tumor tissue itself. Such as, ETBF in the core region of the tumor can affect immune efficacy by recruiting MDSCs into the TME thereby inhibiting CD8^+^ T cell function (132). On the other hand, the gut, as the primary habitat of the microbiota, can influence the TME at distant sites through systemic inflammation and distal signaling when its ecological balance is disrupted. It has been found that gut microbiota imbalance promotes neutrophil enrichment and inflammation in the TME of the primary focus, synergistically promoting peritoneal metastasis of CRC (133).

In addition, the gut microbiota regulates bile acid metabolism through the gut-hepatic axis, which not only influences systemic energy balance but also generates specific bile acid metabolites. These metabolites can directly act on immune cells within the TME, modulating their metabolism and function, thereby impacting tumor immune surveillance (134, 135). At metastatic sites, signals of gut microbiota origin can further remodel the local microenvironment. For example, the translocation of multiple taxa from the CRC gut microbiota, including Bacteroides distasonis and Proteus mirabilis, may be associated with a reduction in hepatic Kupffer cells, thereby compromising liver immune surveillance and creating a more permissive immunosuppressive environment for the colonization of CRC liver metastases (136). Although the gut microbiota regulates the TME through multidimensional mechanisms such as epigenetic modifications, immune cell differentiation and metabolic pathway reprogramming, which is expected to provide individualized flora intervention strategies for cancer therapy. However, it is important to note that the mechanistic pathways involving specific metabolites, as delineated in many studies, may not fully align with the human *in vivo* context due to potential differences in metabolite concentration, receptor expression, and microenvironmental complexity. Therefore, these mechanisms must be rigorously validated in human samples before they can be directly translated into clinical intervention targets.

### 3.3 Relationship between gut microbiota and response to treatment

The role of the gut microbiota in the therapeutic response to CRC has received increasing attention in recent years (125). The influence of the gut microbiota on the response to chemotherapy is mainly reflected in both metabolic regulation and signaling pathway activation. It can convert inactive chemotherapeutic drug precursors into active forms via metabolic enzymes or modulate drug sensitivity via metabolites. A reduced abundance of Clostridium butyricum in patients with CRC lead to decreased sodium butyrate production, which in turn weakens the inhibition of histone deacetylase, ultimately increasing resistance to 5-fluorouracil chemotherapy (137). The dysbiosis caused by Clostridioides difficile infection leads to a deficiency in short-chain fatty acids and an accumulation of secondary bile acids. This accumulation exacerbates chemotherapy resistance in CRC patients by inducing ROS-mediated DNA damage and activating the EGFR-MAPK pathway (138). Fn has been reported to directly promote resistance to 5-fluorouracil in CRC cells by activating the TLR4/MyD88 signaling pathway, and targeting this mechanism, the development of nanomedicines specifically aimed at clearing Fn has emerged as a promising strategy to reverse such drug resistance (139, 140).

During immunotherapy, the composition of the gut microbiota is one of the factors influencing the efficacy of ICIs. The core of this influence lies in the ability of the microbiota to modulate the balance and function of immune cells within the TME (141). For example, a higher abundance of Faecalibacterium prausnitzii correlates with improved response to immunotherapy, a phenomenon that may be associated with an increase in tumor-infiltrating lymphocytes and a reduction in MDSCs (142). More significantly, recent clinical trials have shown that a low-abundance of Actinobacteriota and Bifidobacterium correlates with high efficacy when fecal microbiota transplantation is combined with PD-1 inhibitors for the treatment of MSS-CRC, suggesting that the dynamics of the gut microbiota composition modulate the immunotherapeutic response to treatment (143). Interestingly, some gut microbiotas traditionally considered carcinogenic may exert a positive influence on immunotherapy under specific circumstances. Yu et al. demonstrated that fecal transplants of high-abundance Fn increased the effectiveness of anti-PD-1 treatment for MSS-CRC (144). These findings underscore the complexity of microbiota-immune interactions, whose effects are likely highly dependent on specific bacterial strains, overall community composition, host immune status, and treatment regimen. They provide a compelling rationale for “microbiota-directed” personalized therapy, yet pose significant challenges: what constitutes an “ideal” microbial profile for which patient? There is currently no consensus. Future efforts will require larger, well-designed clinical trials integrated with deep multi−omics analyses to identify more predictive and therapeutically relevant microbial signatures, along with rigorous assessment of the long−term safety of interventions such as fecal microbiota transplantation.

## 4 EV-mediated interaction network of gut microbiota and TME

Within complex biological systems, intercellular communication extends beyond eukaryotic cells. Prokaryotes, including bacteria within the gut microbiota, also actively participate by releasing nanosized particles known as bacterial outer membrane vesicles (OMVs). However, these bacterial vesicles differ fundamentally from eukaryotic EVs, which are generated via the endosomal system or multivesicular body pathways, in terms of biogenesis (direct budding from the outer membrane), core molecular composition (enriched with pathogen-associated molecular patterns such as lipopolysaccharide), and primary functions (toxin delivery and interbacterial competition) (145, 146). The central aim of this review is to delineate how CRC-derived EVs, serving as pivotal signaling vehicles modulated by the gut microbiota, orchestrate the TME through long-range regulation. CRC-derived EVs constitute a high-order, programmable communication system that not only integrates complex biochemical signals from the microbiota but also precisely delivers them to target cells within the TME, thereby playing a significant role in the dynamic regulation of the CRC ecosystem. Host cell-derived EVs enter the gut lumen and systemic circulation and, equipped with a stable lipid bilayer and an ability to cross biological barriers, function as a key communication medium enabling trans‑boundary crosstalk between the intestine and remote TMEs. It was found that the gut microbiota synergistically promotes CRC progression and metastasis through metabolites and immunomodulatory signals that induce chronic inflammation, disrupt intestinal barrier function, and through EV-mediated miRNA delivery (147, 148). Meanwhile, CRC-derived EVs act as molecular messengers for intercellular communication, carrying tumor-specific nucleic acids, proteins and lipid metabolites, thereby remodeling HSCs and regulating the metabolic reprogramming of immune cells, and providing “soil” for distant liver metastasis (73, 149). Consequently, this constructs a dynamic and systematic network of bidirectional regulation.

### 4.1 Gut microbiota regulates secretion of CRC-derived EVs

The ecological composition of the gut microbiota serves as an upstream determinant that regulates the biogenesis of EVs from host CRC cells. This regulation operates at two distinct levels: it exerts a global influence on the overall flux of EV secretion, and it specifically “programs” the molecular cargo composition of EVs. It is increasingly recognized that the gut microbiota can indirectly influence TME remodeling by modulating EVs secretion from host cells (26).Liu et al. demonstrated that intestinal dysbacteriosis promotes exosome secretion in CRC xenograft tumor and leads to poor prognosis in mice by constructing a mouse model, and that the exosome inhibitor, neticonazole, can inhibit tumor growth if administered (150). At a more specific “programming” level, particular pathogenic bacteria and their metabolites can direct host cells to produce cargo-specialized EVs. Moreover, it has been found that P. micra enhances cellular secretion of miR-218-5p-rich exosomes and inhibits PTPRR protein expression, which in turn activates the Ras/ERK/c-Fos signaling pathway driving CRC progression (151). In addition, ETBF promotes the development of CRC by suppressing the expression of exosomal miR-149-3p, and the levels of exosomal miR-149-3p in the plasma of inflammatory bowel disease and CRC patients are negatively correlated with ETBF abundance (152).

The molecular mechanism underlying this regulation orchestrates through multiple synergistic pathways, which may include: direct bacterial attachment or invasion that activates oncogenic signaling pathways such as Wnt/β−catenin and NF−κB in host cells; gut microbiota−derived metabolites (butyrate, secondary bile acids, succinate) acting as signaling molecules or epigenetic modifiers to reprogram host gene expression; and bacterial components triggering host inflammatory responses via pattern−recognition receptors like Toll−like receptors (153–156). Additionally, the interplay between chronic inflammation, the gut microbiota, and EVs is epitomized in colitis-associated CRC, which arises from a background of persistent, dysregulated mucosal inflammation in inflammatory bowel disease (IBD) (157–159). This setting provides a fundamentally distinct “soil” compared to sporadic CRC, characterized by a pervasive inflammatory microenvironment that likely reprograms EV biogenesis and cargo loading from intestinal epithelial and immune cells (160). Inflammation-driven EVs may be enriched in damage-associated molecular patterns, pro-inflammatory cytokines and specific miRNAs that not only perpetuate tissue damage and repair cycles but also actively shape a dysbiotic microbiota and educate the immune system towards tolerance, creating a self-reinforcing vicious cycle that fuels carcinogenesis (161, 162). EVs in IBD have been shown to carry unique molecular signatures that modulate fibroblast activation and barrier integrity, contributing to inflammation persistence (163).

Comparing EV signatures between colitis-associated CRC and sporadic CRC, it may reveal inflammation-specific EV cargos (e.g., proteins or miRNAs associated with NF-κB or STAT3 signaling) that drive tumorigenesis independently of the canonical adenoma-carcinoma sequence, and identify unique biomarkers for risk stratification in high-risk IBD populations (164–166). However, the specific role of the gut microbiota-EV axis in the progression from chronic colitis to cancer remains significantly under-explored. Current evidence is often fragmented, derived from separate studies on IBD inflammation, CRC-derived EVs, or dysbiosis; this makes it difficult to establish direct causal links within the unified framework. The profound influence of the inflammatory milieu itself on both microbiota composition and host cell secretion poses a significant challenge in distinguishing EV cargo that actively drives carcinogenesis from those that are mere bystanders of inflammation. Therefore, investigating the specific modulation of EV secretion and function by the gut microbiota within the IBD context is not only crucial for deciphering the pathogenesis of colitis-associated CRC, but also provides the theoretical groundwork for developing novel biomarkers and intervention strategies for high-risk populations. It is noteworthy that not all gut microbiota−derived signals exert pro−tumorigenic effects on EVs, and most evidence originates from *in vitro* cellular assays or specific animal models. Furthermore, interpreting these findings requires caution, as such studies often employ high bacterial doses or purified metabolites, or rely on cell lines with limited genetic diversity—conditions that may differ substantially from the complex, low−dose, and chronic microbiota−host interactions *in vivo*. Consequently, attributing discrete biological functions, such as pro−metastatic activity, to a specific EV subpopulation remains technically challenging.

### 4.2 EVs mediate gut microbiota-TME communication

EVs serve as key carriers of communication between the gut microbiota and the TME, playing an important role in CRC progression by transmitting bioactive molecules. The core of this process lies in the ability of EVs to serve as stable molecular vehicles, which are transported via the circulatory system and selectively taken up by specific target cells. On one hand, CRC−derived EVs can deliver complex molecular instructions that systemically remodel the microenvironment of distant organs, thereby paving the way for metastasis. For example, CRC-derived EVs induce HSCs to transform into CAFs by carrying TGF-β1, and cooperate with chemokines such as CXCL12 to recruit MDSCs, thereby pre-establishing an immunosuppressive pre-metastatic niche in the liver (167). This illustrates the capacity of EVs to act as a “long−range advance team” for the tumor, capable of pre−modifying distant “soil” before the arrival and colonization of tumor cells. This cross-organ information transfer not only affects local immune responses, but also acts on distant organs through the circulatory system, creating conditions for the formation of PMN (168). EVs derived from CRC can directly participate in signal transduction between tumor cells and other host cells, and indirectly influence the immunosuppressive characteristics of the TME by regulating the composition of the gut microbiota and its metabolic activities. Nevertheless, precise quantitative assessments are currently lacking, and the formation of the pre-metastatic niche is a dynamic, multi-step process involving multiple contributing factors. Thus, a more objective perspective is to establish EVs as an important player in this process, rather than the sole determinant.

**Figure 3:**
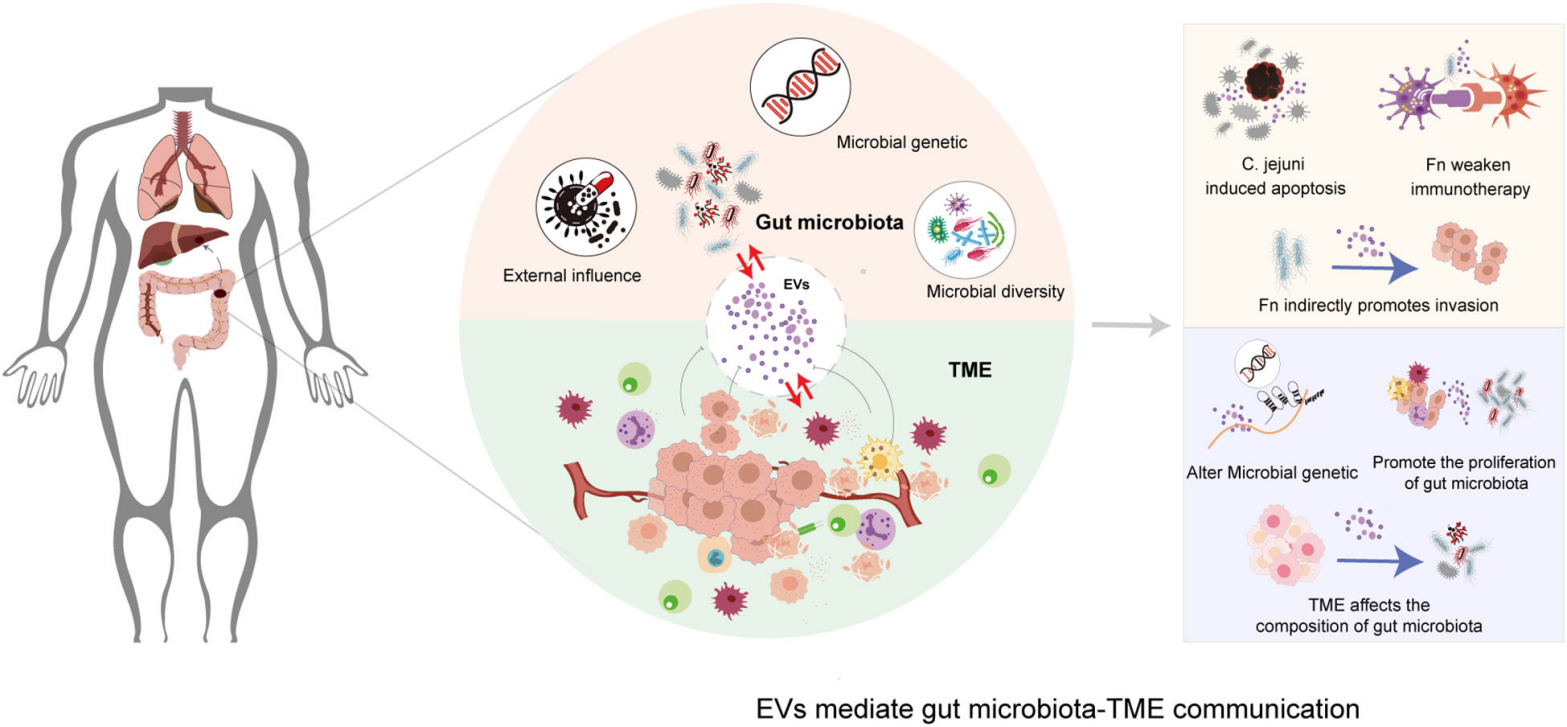
EVs form a critical bidirectional axis for gut microbiota-TME crosstalk: promote tumor invasion and drug resistance signals and influence the genome and composition of the gut microbiota in CRC.

On the other hand, these EVs serve as substantive carriers that transmit signals from the gut microbiota. For example, the infection of Fn can induces CRC cells to secrete exosomes rich in miR-1246/92b-3p/27a-3p and CXCL16, promoting tumor invasion and metastasis (169). EVs play a central role in tumor metastasis and immune evasion by mediating complex interactions between the gut microbiota and the TME (**Figure 3**). EV−mediated communication extends beyond mere signal transduction to the remote execution of functions, enabling the relatively independent gut microbial ecosystem to exert sustained and profound regulatory influence over distant tumor progression. Future research could focus on developing precise intervention strategies targeting EVs, combining gut microbiota regulation with clinical CRC treatment to provide new insights for improving patient outcomes. Despite its theoretical appeal, the operational efficiency, relative contribution, and interplay of this axis with other communication routes within the authentic human TME must be further substantiated and delineated through more rigorous *in vivo* tracing, cell−specific EV−depletion models, and advanced molecular quantification techniques.

### 4.3 EVs and gut microbiota synergistically reshape the TME

The regulation of EV secretion from CRC cells by the gut microbiota lies not only in dynamic metabolic and epigenetic crosstalk but also in their combined role as an integrated signaling and execution network. On one hand, gut microbiota-derived metabolites achieve functional “hitchhiking” and precision delivery via host EVs to cooperatively remodel the TME. These metabolites are actively taken up by host cells and encapsulated into their secreted EVs, gaining protection from the lipid bilayer for stable transit and targeted delivery (170–173). Fang et al. found that succinate, a metabolite of Fn, can enter host cells via EVs and inhibit the cGAS-IFNβ signaling pathway, leading to downregulation of CCL5 and CXCL10 expression, thereby hindering the infiltration of CD8^+^ T cells into the TME and ultimately weakening the efficacy of anti-PD-1 immunotherapy (174). Similarly, EVs secreted by CRC cells may carry antimicrobial peptides, immunomodulatory proteins, or metabolic enzymes, which can directly suppress the proliferation of beneficial bacteria or promote the expansion of pathogenic bacteria, and potentially influence additional types of immune cells. Fn-derived succinic acid, after being encapsulated by host EVs, promotes the recruitment of MDSCs to form PMN by inhibiting NKG2D receptor expression and cytotoxic function in NK cells (167).

On the other hand, engineered intervention strategies based on this cooperative network are being conceptually validated for their therapeutic potential, while in turn revealing the operational logic of the network itself. Researchers have developed the iMASSAGE platform, which uses wireless control to release mechanical stimulation signals from hydrogels, directing macrophages to produce high doses of EVs for regulating the gut microbiota and inhibiting the progression of CRC (175). Interventions targeting the origins of collaborative networks have also demonstrated therapeutic efficacy. Research has found that high levels of Fn can upregulate VEGF expression at the CRC tumor site via EVs, inducing angiogenesis, and inhibit anti-angiogenic therapy by recruiting M2-type macrophages. However, targeted delivery of antimicrobial agents and anti-angiogenic genes to clear Fn and inhibit angiogenesis can reverse the immunosuppressive microenvironment (176). This suggests that disrupting the detrimental synergy between the microbiota and EVs may represent a viable therapeutic strategy. Through metabolite hitchhiking, signal integration, and functional cooperation, host EVs and the gut microbiota constitute a tightly coupled consortium that remodels the TME. Moving forward, research must develop precisely targeted combination therapies that simultaneously address the microbiome and host cell communication networks, while rigorously distinguishing the cellular origins of vesicle populations.

### 4.4 Limitations of the bidirectional interaction model

Based on current evidence, a working model emerges of a complex, EV-mediated bidirectional circuit connecting the gut microbiota and the TME. Its core is the established “Gut microbiota-CRC-derived EVs-TME” axis, while acknowledging the plausible reverse direction in which TME or systemic inflammation may alter host EV profiles and subsequently impact microbial ecology. Direct proof for this feedback is still lacking, yet pursuing it will deepen the ecological perspective of CRC as a systemic disorder.

Despite the momentum and promise, the significant limitations of present studies must be soberly acknowledged. First, direct *in vivo* evidence and causal confirmation remain insufficient. Many reported molecular mechanisms originate from *in vitro* co−culture studies or employ high−dose, heterogeneous EV preparations. Under physiological concentrations and within the complex multicellular milieu *in vivo*, the precise biodistribution, uptake kinetics, and relative functional contributions of specific EV subtypes require rigorous validation using advanced techniques such as real−time intravital imaging and cell−specific EV reporter systems. Secondly, the tissue and cellular specificity of signaling pathways is poorly defined. Whether gut microbiota−mediated regulation of EVs and subsequent EV−driven effects on the TME vary according to CRC anatomical location, molecular subtype, or individual host genetic background is largely unknown, such as specificity is fundamental to developing precision therapeutic strategies. Moreover, current research predominantly focuses on pro−tumorigenic networks, while the possibility that specific probiotics may induce the release of host EVs with anti−tumor activity remains severely under−explored. In addition, direct experimental evidence for the reverse communication pathway is notably scarce, limiting our understanding of the full dynamic interplay within this ecosystem. Finally, the field continues to be constrained by the inherent heterogeneity of EVs and the lack of methodological standardization in isolation and characterization, which compromises the comparability and reproducibility of findings across studies and poses a major obstacle to constructing a unified model. Future research must urgently address these gaps by employing more sophisticated models and technologies, thereby translating this systems−level insight into precise, effective clinical interventions that can improve outcomes for CRC patients.

## 5 Current status of clinical applications

As a key component of liquid biopsy, EVs offer distinct advantages in the clinical management of CRC, including non−invasive sampling, the ability to enable dynamic monitoring, and providing a real−time snapshot of the TME. The exploration of EV−based applications in CRC therapy is also rapidly diversifying, encompassing cutting−edge areas such as immune modulation, targeted drug delivery, and microbiota regulation (28, 177, 178). However, translating these promising scientific discoveries into widely applicable clinical tools still faces a series of formidable challenges, ranging from technical standardization to clinical validation.

### 5.1 Development of diagnostic biomarkers

Non-invasive liquid biopsy based on EVs in CRC has attracted considerable attention due to its potential in early diagnosis and prognosis assessment. Exploring the interaction between CRC-derived EVs and the gut microbiota paves the way for developing novel diagnostic biomarkers with enhanced specificity and sensitivity (177, 179). Research has found that the expression of transmembrane proteins CD147 and A33 in EVs from fecal samples directly contacting the intestinal mucosa in CRC patients was significantly higher than in healthy individuals, and highly consistent with expression levels in cancerous tissue, and these proteins also demonstrate high sensitivity and specificity in independent cohorts, providing new insights for non-invasive screening of CRC (180, 181). With advances in isolation and detection technologies, high-performance diagnostic models based on EVs are continuously emerging. For example, using hydrophobic interaction for efficient capture of plasma EVs combined with data-independent acquisition proteomics, a ColonTrack model consisting of CTTN, HNRNPK, and PSMC6 was screened out, with an AUC of 0.97 or higher for early CRC, significantly superior to the traditional marker CEA (182). Similarly, the application of machine learning to serum exosomal proteomic profiling enabled the development of a diagnostic model based on key proteins like platlet factor 4 and acetyl-CoA C-acetyltransferase - a model that achieved an AUC exceeding 0.960 and surpassed the performance of CEA and CA199 (183). More significantly, the molecular information carried by EVs can be correlated with gut microbial community characteristics. The study revealed that CRC cell-derived exosomes selectively carry certain miRNAs whose levels correlated positively with Fn abundance in CRC tissues (169). It study has elucidated a pivotal interaction network comprising 22 lncRNAs, 14 mRNAs/proteins, and 9 metabolites, proving its essential function in CRC progression and immunomodulation (184). This suggests that EV-carried miRNAs and lncRNAs can influence the TME by modulating gut microbiota-host interactions, and also hold significance for biomarker development and the construction of machine learning models (84, 185).

However, beneath this optimistic outlook, it is crucial to soberly recognize the severe challenges facing the clinical translation of EV−based biomarkers. First, tumor specificity remains a central hurdle: how to specifically identify and enrich the minute EV signals derived from CRC against the overwhelming background of EVs released by normal tissues into the circulation. Second, pre−analytical variables and standardization represent a major source of variability: every step—from the collection, transport, and storage of blood or stool samples to EV isolation, purification, and nucleic acid/protein extraction—can dramatically affect the final assay outcome, and a globally unified, standardized operating procedure is currently lacking. Third, validation bottlenecks are widespread: many studies reporting high−performance diagnostic signatures are based on small−scale, retrospective case−control cohorts, which may overestimate diagnostic efficacy. To advance such markers toward clinical use, they must undergo validation in large−scale, prospective, multicenter clinical trials to confirm their reliability, reproducibility, and cost−effectiveness in real−world screening or diagnostic settings. Overcoming these challenges therefore constitutes an essential pathway for moving EV−based liquid biopsy from the laboratory to the clinic.

### 5.2 Treatment strategies

EVs are an ideal platform for delivering various therapeutic molecules due to their inherent biocompatibility and low immunogenicity (186). More importantly, based on the understanding of the core role of EVs in mediating chemotherapy resistance and immunosuppression, targeting EVs has become a critical component in designing next-generation integrated treatment strategies (187). Engineered modification or targeted intervention of EVs can be used as therapeutic carriers and synergize with the gut microbiota to provide new insights into reversing immune-suppressive TME (102, 188). Engineered EVs refer to vesicular systems in which natural EVs are modified through biological, chemical, or genetic engineering approaches to enhance their targeting specificity, cargo-loading capacity, or therapeutic efficacy, thereby transforming them into biological tools capable of precise drug delivery (189, 190). Compared to conventional synthetic carriers, the superior properties of EVs have opened up new avenues (191). With the continuous advancement of EVs engineering technology, it is anticipated that a growing number of innovative therapeutics will receive both approvals and market authorization from regulatory agencies in the future (192). It has been reported that stimulating macrophages via physical or biological methods can induce them to produce abundant regulatory EVs. In models of colitis−associated cancer, these EVs have shown efficacy in ameliorating gut dysbiosis and suppressing tumor progression, thereby providing proof−of−concept for developing controllable EV “bio−factories” (175). Beyond their role as delivery vehicles, engineered EVs can be precisely designed to function as “decoys” or modulators, neutralizing tumor-derived immunosuppressive EVs or directly intervening in immune checkpoint pathways (193). However, alongside the recognized potential of engineered EVs, serious translational challenges must be addressed. Most work is still preclinical, and advancing to a reliable therapy demands solutions to key bottlenecks spanning scalable GMP manufacturing, efficient and reproducible cargo loading, controlled *in vivo* targeting, and long−term safety. These issues collectively sustain a formidable translational gap between bench−side optimism and bedside utility.

While the gut microbiota shapes CRC-derived EVs via metabolic and immune cues, engineered EVs conversely offer a tool to reprogram the gut niche. This suggests a bidirectional therapeutic future. On one hand, engineered EVs could be designed to enhance the intestinal targeting and stability of EVs by loading them with butyric acid and IPA through electroporation or ultrasound (148, 194). On the other hand, oral administration of specific probiotics may stimulate host intestinal epithelial cells to secrete EVs with anti−inflammatory or immune−activating functions, thereby indirectly modulating systemic immune responses (175). A more direct approach targets the oncogenic bacteria themselves. For instance, developing nanomedicines capable of clearing Fn could cut off the source of its induced pro−tumorigenic EV production, thereby reversing the associated immunosuppressive and pro−angiogenic effects (176). It is notable that intestinal microbiota dysbiosis may indirectly undermine chemotherapy-based immune priming or exacerbate immune checkpoint-mediated suppression by altering the cargo of EVs. This provides a direction for jointly targeting the gut microbiota-EVs axis to improve the efficacy of both conventional and immunotherapeutic regimens (125). However, such ecology−based modulation strategies are equally fraught with complexity. The vast inter−individual variation in gut microbiota composition makes it difficult to design standardized interventions; the efficiency of oral or intestinal delivery of EVs or related agents is impeded by the harsh gastrointestinal milieu; furthermore, the long−term ecological consequences and safety of microbiota manipulation still necessitate thorough evaluation in rigorous clinical trials. Therapeutic resistance in CRC originates from an adaptive system comprising tumor cells, the immune microenvironment, and intercellular communication (28). Therefore, the most promising direction is to develop integrated therapeutic strategies that simultaneously dismantle the multiple defense mechanisms of this system. For example, combining capecitabine-based chemotherapy with inhibitors that target EV function can block EV-mediated adaptive immunosuppression, thereby fully harnessing the immunostimulatory potential of chemotherapy to enhance therapeutic efficacy (195). In CRC, particularly the MSS type, both targeting CTLA-4 and PD-1/PD-L1 must contend with a more complex immunoregulatory barrier (196, 197). Future clinical trials need identify the patient population most likely to benefit from a multimodal combination regimen involving chemotherapy, immune checkpoint inhibitors, EVs, and gut microbiota modulation in CRC, thereby enabling the personalized design of critical parameters such as treatment timing and drug dosage. This integrated strategy, grounded in systems biology, holds promise not only for overcoming current therapeutic resistance but also for significantly expanding the population eligible for and benefiting from immunotherapy.

In summary, whether serving as engineered drug carriers or as tools to intervene in the gut microbiota−TME communication network, EV−based therapies face common and formidable translational bottlenecks on the path to clinical application. First, manufacturing and pharmacokinetic hurdles are prominent: how can therapeutic−grade EVs be produced at scale with high efficiency, cost−effectiveness, and reproducibility? How can their *in vivo* distribution be precisely controlled to achieve targeted enrichment in tumor tissues while effectively traversing complex physiological and pathological barriers? Second, safety and immunogenicity concerns cannot be overlooked: although natural EVs exhibit low immunogenicity, could engineering modifications trigger unforeseen immune responses or off−target toxicity? Is there a risk of rejection for allogeneic EV sources? These questions necessitate extensive long−term preclinical toxicology studies and early−phase clinical trials. Finally, the challenge of personalized therapy arises from the high heterogeneity of CRC, suggesting that a “one−size−fits−all” EV therapy may have limited efficacy. Future success will likely depend on integrating EV−based interventions with personalized biomarkers—such as tumor molecular subtype, patient−specific gut microbiota profiles, and plasma EV molecular signatures—to enable precise matching to specific patient subgroups (169, 198). Consequently, although EVs offer an exciting new paradigm for CRC treatment, their ultimate successful translation will depend on interdisciplinary collaboration to tackle the full spectrum of challenges, spanning fundamental biology, materials engineering, and clinical development.

### 5.3 Future challenges

The revolutionary potential of EVs in the diagnosis and treatment of CRC is undeniable. Yet, their successful translation and widespread adoption in clinical practice continue to face a series challenges (199, 200). These challenges are rooted in the biological intricacy of EVs and span the full pathway from fundamental discovery to therapeutic development.

The primary and fundamental challenge lies in the standardization of methodologies and the elucidation of EV heterogeneity. Most preclinical studies employ heterogeneous populations of EVs obtained by conventional methods such as differential ultracentrifugation, size-exclusion chromatography, or polymer-based precipitation (45–49). While these “mixed preparations” have advanced mechanistic understanding, they also complicate the precise attribution of specific biological functions to individual EV subtypes and substantially compromise the comparability and reproducibility of findings across different laboratories. Although advances in separation technology are enabling new methods that allow for the selective isolation of specific EV subpopulations. Building upon traditional techniques, developing and integrating these novel pathways offers a route to markedly improved isolation efficiency and specificity (201). Such as, the application of DNA computing-mediated microfluidics enables the successful isolation of specific EV subpopulations that co-express both EpCAM and PD-L1 (202). Meanwhile, methods based on covalent chemistry and click chemistry achieve effective isolation of EVs by exploiting the heterogeneity of membrane proteins (203, 204). And aptamer-based subpopulation separation methods enable reversible release and non-destructive isolation of EV subpopulations, making them suitable for downstream applications (202, 205). However, these technologies themselves remain immature and suffer from a lack of unified operational and quality−control standards. With the growing appreciation of EVs heterogeneity, their efficient isolation and characterization have become pivotal for ensuring research reproducibility and clinical translation potential (206). Therefore, establishing a widely accepted set of standard operating procedures for EV isolation, characterization, and quantification—applicable across different contexts (discovery research vs. diagnostic testing vs. therapeutic products). This necessitates developing highly specific techniques for isolating EV subpopulations, establishing standardized protocols that meet clinical practice requirements, and validating them through large-scale, multi-center studies, ultimately enabling the widespread application of EVs in the early diagnosis, treatment response monitoring, and prognostic assessment of CRC.

Secondly, the scalable manufacturing, stability, and *in vivo* pharmacology of therapeutic EVs confront severe engineering bottlenecks (192). The “functional plasticity” of EVs is a double-edged sword: it endows EVs with the potential to respond to the microenvironment and execute complex tasks, while also making their *in vivo* behavior difficult to predict and control precisely (39, 207). The high heterogeneity of CRC requires EV therapies to be combined with individualized biomarkers, such as gut microbiota composition, which can serve as a predictive indicator of EV intervention efficacy (198). Currently, there is no standardized separation and purification technology for EVs. Although research has developed the DSPE-Beads method to efficiently capture EVs through hydrophobic interactions, increasing protein coverage by 40%, it is not suitable for standardization due to its high cost in large-scale production (208). On the manufacturing front, achieving the large−scale, high−reproducibility, GMP−compliant production of therapeutic−grade EVs constitutes a central obstacle to industrialization. This encompasses the selection of stable cell lines, serum−free culture processes, efficient yet gentle isolation and purification methods, loading techniques with high drug–encapsulation efficiency, and optimized long−term storage solutions such as lyophilization (209). On the delivery front, while engineering (e.g., adding targeting ligands) can enhance specificity, the pharmacokinetics (distribution, metabolism, half−life), immunogenic potential, and potential off−target effects of modified EVs remain insufficiently studied. Unlike synthetic nanoparticles with defined composition and fixed structure, the composition and membrane properties of EVs secreted by living cells exhibit inherent batch−to−batch variability, complicating quality control and assurance.

Thirdly, a challenge lies in deciphering the overwhelming complexity of the gut microbiota–TME network itself. The interactions are highly heterogeneous, context-dependent, and non-linear, making it difficult to extract dominant drivers and robust biomarkers from single-molecule studies. Future research must embrace integrative systems biology approaches, involving the concerted analysis of multi-omics data layers, including EVs, host tumor and immune cell transcriptomes, gut microbiota, and metabolic profiles, which derived from the same patient cohorts (210). In this context, tools like WGCNA offer a powerful paradigm. WGCNA can identify clusters of highly co-expressed genes or molecules across these multi-omics datasets (211, 212). By correlating these modules with specific clinical features (e.g., inflammation grade, immune suppression, chemotherapy resistance), researchers can pinpoint EV-associated molecular modules that are central to network function (165, 183). This module-centric strategy provides several key advantages, which enhances robustness against the noise inherent in low-abundance EV cargo, prioritizes pathway-level dysregulation over individual biomarkers, and reveals the core regulatory networks that drive phenotypic outcomes (213). It provides the rational foundation for combining EV research with machine learning and network-based modeling, thereby guiding the development of targeted interventions within this pathogenic network.

Finally, successful clinical translation urgently requires addressing the dual challenges of safety validation and personalized strategy. Safety must be paramount for any novel therapy. For EV-based therapies, especially those involving engineering or allogeneic sources, rigorous preclinical and clinical trials are essential to systematically evaluate their long-term toxicity, risks of immune activation or suppression, and potential impacts on the host microbiota (178, 214). CRC and its microenvironment exhibit high inter-patient heterogeneity, meaning that a single-mode EV therapy will likely benefit only a subset of patients. Consequently, future development must inevitably trend toward “personalization”. This necessitates not only optimizing the EV product itself but also establishing a predictive biomarker framework. Patient-specific features such as gut microbiota profiles, tumor molecular subtypes, and plasma EV molecular signatures may serve as key indicators for predicting responses to specific EV interventions—whether as diagnostic tools, drug carriers, or ecological modulators (198, 215). Only by integrating EV therapy with precise patient stratification can its therapeutic potential be maximized and non-effective treatment avoided. In conclusion, while the prospects for EVs in CRC are undoubtedly bright, the path to clinical translation remains arduous. Overcoming these challenges demands close and seamless collaboration among biologists, engineers, clinicians, and regulators, requiring sustained, concerted endeavors across multiple frontiers, including standardization, manufacturing science, translational pharmacology, and precision medicine.

## 6 Conclusion and prospects

As a highly prevalent malignant tumor worldwide, CRC is closely related to the complex interactions between the TME and the gut microbiota, and its research focus is shifting from analyzing localized lesions to examining the tumor as an integrated ecosystem. This review systematically elucidates the central hub role of CRC-derived EVs in integrating the bidirectional dialogue between the gut microbiota and the TME. Substantial evidence indicates that CRC-derived EVs are not passive metabolic by-products but are dynamic information carriers actively “programmed” by signals from both tumor cells and the microbiota. EVs not only directly participate in tumor immune escape and the construction of the PMN, but also indirectly reshape the TME by regulating the gut microbiota and its metabolites, forming a bidirectional regulatory network. Through the delivery of specific biomolecules, they orchestrate key pro-tumor processes including immunosuppression, angiogenesis, metastasis, and drug resistance. Furthermore, the gut microbiota shapes this activity by reprogramming EV biogenesis and cargo, converting local microbial cues into systemic pathogenic signals. This axis, which positions EVs as both signal executors and transducers, provides a foundational framework for ecologically-informed therapeutic strategies that target the TME as a whole.

Nevertheless, clinical translation is hindered by fundamental challenges arising from biological complexity. A primary gap is mechanistic: current data are largely correlative. Key unresolved questions include the exact molecular switches linking microbial signals to EV biogenesis and the quantitative parameters governing EV targeting and cargo release *in vivo*. Secondly, in terms of the clinical relevance of the research model, most studies are limited to animal models or *in vitro* experiments, with insufficient clinical translational evidence, and their safety and long-term efficacy still need further verification. The heterogeneity of EVs and the specificity of target sites still require systematic analysis. Consequently, the functional pathways of EVs validated in these models require rigorous re-evaluation in both preclinical and clinical settings to confirm their dominant role and targetability within the authentic tumor ecosystem of patients. Finally, at the level of technological translation and personalized application, an intrinsic trade-off exists between the inherent heterogeneity of EVs as natural nanocarriers and the uniformity demanded of standardized drugs or diagnostic tools.

In future, research should advance this field along a synergistic pathway from understanding mechanisms to achieving clinical benefits. This advancement hinges on a dual strategy: technological optimization and systems-level decoding. First, we can combine gene editing and membrane engineering technologies to optimize the targeting of EVs. Second, it is imperative to develop multi-omics integration platforms to decode the dynamic EVs and gut microbiota–TME interactome. Tools like WGCNA can integrate data and identify functionally cohesive molecular modules that are mechanistically pinpointed the most therapeutically vulnerable nodes in the network. It provides the rational foundation for developing precise biomarkers and target for high-risk populations (165).

To this end, it will be essential to utilize single-cell sequencing, spatial transcriptomics, and metabolic analysis technologies to reveal the sorting of EV contents, metabolic reprogramming of the gut microbiota, and immune remodeling of the TME. Clinical translation research must adopt a pragmatic, stepwise strategy from the outset. This involves establishing high−quality prospective longitudinal biobanks linked to comprehensive clinical databases, which systematically collect multi−dimensional data (tissue, blood, stool, and clinical information) from CRC patients before and after therapy. Thereby enabling the mapping of the dynamic evolution of the gut microbiota−EVs−TME network in a real−world context and the identification of integrated biomarkers capable of predicting treatment response or prognosis. Building on this foundation, rationally designed early−phase clinical trials should be advanced. And focus on the innovation of personalized treatment strategies, and design combination treatment plans based on patient gut microbiota characteristics, EVs molecular profiles, and the TME changes. For example, engineered EVs loaded with gut microbiota metabolic inhibitors or immune checkpoint blockade antibodies can achieve “dual-targeted” treatment of CRC. The core objective of such trials is not only to assess safety, but also to validate the principles of this ecosystem-targeting theory in humans and to identify the patient subpopulations most likely to benefit.

In summary, CRC-derived EVs, as a key mediator connecting the gut microbiota and TME, hold promise for overcoming the current treatment bottlenecks of CRC and developing precise intervention strategies in the future. These studies have deepened our understanding of tumor immune escape mechanisms and provided new insights into overcoming current treatment bottlenecks. By synergizing rigorous mechanistic dissection, innovative technology integration, and safe clinical exploration through interdisciplinary collaboration, a new path can be forged to improve the prognosis of CRC, ultimately achieving the leap from mechanistic discovery to clinical application.

## Glossary

CRC: Colorectal cancer
EVs: Extracellular vesicles
TME: Tumor microenvironment
LC3: Microtubule-associated protein 1 light chain 3
MDSCs: Myeloid-derived suppressor cells
CTLA-4: Cytotoxic T-lymphocyte-associated protein 4
TLR2-MYD88: Toll-like receptor 2-Myeloid differentiation primary response protein 88
Rab: Ras-like proteins in brain
GTPase: Proteins are small guanosine triphosphatase
SNARE: Soluble N-ethylmaleimide-sensitive factor attachment protein receptors
PTEN: Phosphatase and tensin homologue
AKT PKB: Protein Kinase B
NF-κB: Nuclear factor kappa-B
PD-1: Programmed death 1
PD-L1: Programmed cell death ligand 1
APCs: Antigen−presenting cells
Tregs: Regulatory T cells
MSS: Microsatellite-stable
HSCs: Hepatic stellate cells
INSIG1: Insulin-induced gene 1
TLR4: Toll-like receptor 4
TGF-β: Transforming growth factor-β
TAMs: Tumor-associated macrophages
EGFR: Epidermal growth factor receptor
LncRNA: Long non-coding RNA
USP22: Ubiquitin-specific peptidase 22
INHBC: Inhibin subunit beta C
APC: Antigen-presenting cell
KRAS: Kirsten rat sarcoma viral oncogene homolog
TP53: Tumor Protein p53
ERK: Extracellular regulated protein kinases
CREPT: Cell-cycle related and expression-elevated protein in tumor
TNFR2: Tumor necrosis factor receptor 2
CDC42: Cell Division Cycle 42
NOD1: Nucleotide Binding Oligomerization Domain Containing 1
SNHG10: Small Nucleolar RNA Host Gene 10
VEGF: Vascular endothelial growth factor
HIPK2: Homeodomain-interacting protein kinase 2
circCOL1A1: circCollagen Type I Alpha 1 Chain
EIF4A3: Eukaryotic Translation Initiation Factor 4A3
PMN: Pre-metastatic niche
PI3K: Phosphatidyl-inositol-3-kinase
EMT: Epithelial-mesenchymal transition
JAK2: Janus kinase-2
STAT3: Signal transducer and activator of transcription 3
IBD: Inflammatory bowel disease
ICIs: Immune checkpoint inhibitors
MFGE8: Milk Fat Globule EGF Factor 8
BRAF: An activating missense mutation in codon 600 of exon 15 (V600E) of B-Raf proto-oncogene
Fn: Fusobacterium nucleatum
BFT: Bifidobacterium fragilis toxins
ETBF: Enterotoxigenic Bifidobacterium fragilis
P. micra: Parvimonas micra
MAPK: Mitogen-activated protein kinase
PMN-MDSCs: Polymorphonuclear myeloid-derived suppressor cells
MSS: Microsatellite-stable
OMVs: Outer membrane vesicles
PTPRR: Protein Tyrosine Phosphatase Receptor Type R
CAFs: Cancer-associated fibroblasts
PD-1: Programmed death-1
MISEV: Minimum information for studies of extracellular vesicles
iMASSAGE: *In-situ* and sustainable generating extracellular vesicles
NKG2D: Natural killer group 2 member D
CTTN: Cortactin
HNRNPK: Heterogeneous nuclear ribonucleoprotein K
PSMC6: Proteasome (prosome, macropain) 26S subunit, ATPase, 6
AUC: Area under the curve
EpCAM: Epithelial cell adhesion molecule
GMP: Good Manufacturing Practice
WGCNA: Weighted gene co-expression network analysis.
